# Rare But Fatal: Hemophagocytic Lymphohistiocytosis (HLH) With Acute Acalculous Cholecystitis

**DOI:** 10.7759/cureus.31737

**Published:** 2022-11-21

**Authors:** Faiza Javed, Jacob Crouch, Ellen Combs

**Affiliations:** 1 Hospital Medicine, University of Kentucky College of Medicine, Lexington, USA; 2 Internal Medicine, University of Kentucky College of Medicine, Lexington, USA

**Keywords:** immuno suppressant, granulomatosis with polyangiitis (gpa), gpa, acute cholecystits, hemophagocytic lymphohistiocytosis (hlh)

## Abstract

Acalculous cholecystitis is an acute inflammatory disease of the gall bladder with high morbidity and mortality rate. It can be seen in trauma, burns, sepsis, total parenteral nutrition, prolonged fasting, and autoimmune diseases. However, there are very few reports of acalculous cholecystitis with macrophage activation syndrome (MAS) and hemophagocytic lymphohistiocytosis (HLH) in patients with underlying rheumatic/autoimmune disorders.

Here we report a 23-year-old male with a past medical history of granulomatosis with polyangiitis who presented with fever, weight loss, and pancytopenia. A comprehensive infectious evaluation was done including bacterial cultures and viral and fungal serologies. Repeat abdominal imaging obtained later due to developing abdominal pain raised concerns for acute acalculous cholecystitis. Despite aggressive management of sepsis, the patient continued to decline clinically. HLH was suspected when the patient was found to meet the clinical criteria with fever, splenomegaly, cytopenia, hypertriglyceridemia, elevated liver function tests, hypofibrinogenemia, and ferritin of 22K ng/mL, absent NK cell activity, and elevated soluble CD25 receptor levels. Bone marrow biopsy did not reveal hemophagocytosis. Intravenous methylprednisolone was started and the patient showed remarkable clinical improvement with a decrease in all inflammatory markers and did not require any surgical intervention. On the review of the literature, we were able to identify four female patients with underlying adult-onset Still’s disease and Kikuchi disease who presented with HLH along with acalculous cholecystitis likely triggered by flare. Our male patient presented with HLH and acute acalculous cholecystitis. He had a history of granulomatosis polyangiitis (GPA) that remained in remission. Hypersecretion of pro-inflammatory cytokines and cytotoxic cells in HLH promotes ischemia of the gall bladder wall. Early initiation of immunosuppressive therapy under careful observation can prevent surgical intervention and mortality in these patients.

## Introduction

Acute acalculous cholecystitis is severe inflammation of the gall bladder seen in critically ill patients with underlying conditions like sepsis, autoimmune diseases, malignancy, burns, trauma, shock, and patients on total parenteral nutrition. The pathogenesis of acute acalculous cholecystitis is a paradigm of complexity. Ischemia and reperfusion injury, or the effects of eicosanoid proinflammatory mediators, appear to be the central mechanism [1]. Acalculous cholecystitis has been described in the past in patients with HLH and macrophage activation syndrome (MAS) with underlying autoimmune diseases. Here we describe a 23-year-old male with a history of granulomatosis with polyangiitis, in remission who presented with symptoms of HLH and was found to have acute cholecystitis. It was unclear, if this cholecystitis triggered HLH in this high-risk patient or if cholecystitis developed as a manifestation of uncontrolled immune activation. However, since the patient failed to improve clinically after being treated with antibiotics, it is suspected that HLH activated a cascade of inflammatory cytokines causing ischemia and damage to the endothelial lining of the gall bladder. We successfully treated this patient by early recognition and started immunosuppressive therapy.

## Case presentation

A 23-year-old male with a history of granulomatosis with polyangiitis with renal involvement (diagnosed at age 16, on prednisone and azathioprine) and stage 4 chronic kidney disease with a baseline creatinine of 3.1 mg/dL initially presented for his fatigue, decreased appetite, and subjective fevers at home. His vitals were significant for fever with lab work showing new pancytopenia, elevated erythrocyte sedimentation rate (ESR) and C-reactive protein (CRP), and elevated ferritin (as shown in Table 1). Peripheral smear showed moderate anemia with mild macrocytosis, elliptocytosis, polychromasia, rare schistocytes, and marked leukopenia with neutropenia. 

**Table 1:** Patient's temperature and laboratory investigations on admission and key hospital days. HD, hospital day; WBC, white blood cell; ANC, absolute neutrophil count; CRP, C-reactive protein; ESR, erythrocyte sedimentation rate; INR, international normalized ratio; aPTT, activated partial thromboplastin time; LDH, lactate dehydrogenase; AST, aspartate transaminase; ALT, alanine transaminase; AlkPhos, alkaline phosphatase

T_max_ and laboratory investigation of case presentation						
	Initial presentation	Cholecystostomy tube placement (HD8)	Steroids initiated (HD10)	Day of discharge (HD15)	Reference range	Units
T_max_	103.1	102.9	101.6	98.2	95.5-99.5	°F
WBC	0.57	1.2	1.83	2.29	3.7-10.3	10^3^/uL
ANC	0.38	0.52	0.9	1.36	1.6-6.1	10^3^/uL
Hemoglobin	7.5	7.2	8.3	7.8	13.7-17.5	g/dL
Platelets	127	34	34	45	155-369	10^3^/uL
CRP	32.9				<8	mg/L
ESR	38				<15	mm/hr
Fibrinogen	457	375	290	203	208-459	mg/dL
INR	1.2	1.1	1.2	1.1	0.9-1.1	
aPTT	48	43	44	29	25-35	sec
LDH	316	747	651	454	116-250	U/L
Triglycerides	151	364	529	519	<150	mg/dL
AST	23	125	65	19	12-40	U/L
ALT	27	123	87	5	11-41	U/L
Albumin	4	3.2	2.8	2.8	3.5-5.2	g/dL
AlkPhos	44	240	187	135	40-115	U/L
Ferritin	2,297	25,820	20,257	7,878	20-400	ng/mL

The patient was noted to be intermittently febrile. Broad-spectrum antibiotics including vancomycin, cefepime, and metronidazole were started. A comprehensive infectious workup was sent. Urinalysis and urine culture were negative. Multiple blood cultures drawn at several days showed no growth. Amylase and lipase levels were normal. Other tests including human immunodeficiency virus (HIV) antibody and antigen screen, hepatitis B surface antigen, hepatitis C antibody, monospot test, cytomegalovirus (CMV) antibody and PCR, galactomannan, beta D glucan, cryptococcal antigen, histoplasma urinary antigen and serum antibody, blastomycoses antibody, *Clostridium difficile* polymerase chain reaction (PCR), Epstein-Barr virus PCR, *Bartonella henselae* immunoglobulin M (IgM) and immunoglobulin G (IgG), *Coxiella burnetii* serology, *Borrelia burdorferi* antibody, *Rickettsia rickettsii* antibody, and *Ehrlichia* PCR were sent and were all found to be negative. CT chest, abdomen, and pelvis did not show any potential nidus of infection. At this point antibiotics were discontinued, and he underwent bone marrow biopsy due to persistent fevers and pancytopenia. It displayed hypocellular marrow (20% cellularity) with no evidence of malignancy or hemophagocytosis. CMV, parvovirus, Fite stain, Gram stain, acid-fast bacilli stain, and KOH were all negative. Aerobic and anerobic cultures, fungal cultures, and mycobacterial cultures did not show any growth. Flow cytometry had absent B cells with no evidence of blasts. Cytogenetics and karyotyping were normal. No hemophagocytosis was present. More specific blood tests were significant for elevated soluble CD25 and critically low NK cell activity. His symptoms were attributed to now hemophagocytic lymphohistiocytosis (HLH). During this period of time, concerns for an active flare of granulomatosis with polyangiitis were low due to stable creatinine, normal proteinase three antibodies, and the absence of hematuria. 

**Figure 1:**
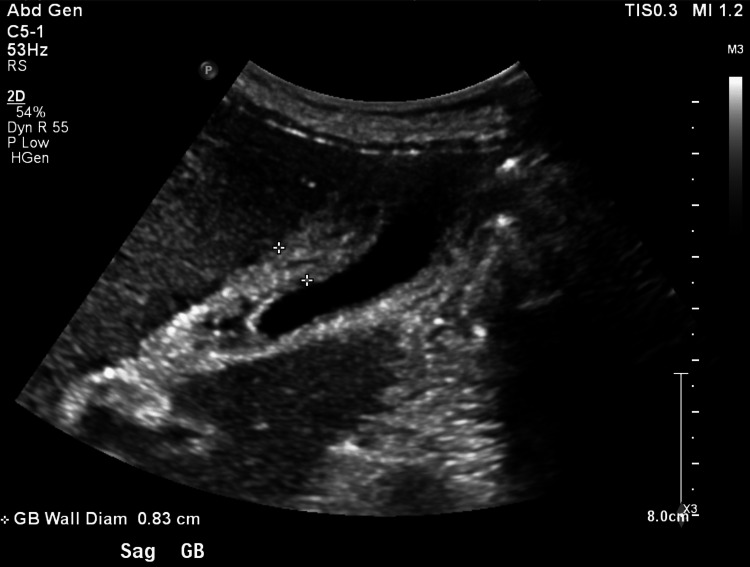
Abdominal ultrasound showing edematous gall bladder with some pericholecystic fluid.

On Day 7, he started to develop right upper quadrant pain. His liver function tests demonstrated elevated alkaline phosphatase, aspartate transaminase (AST), alanine transaminase (ALT), and hypoalbuminemia. Abdominal ultrasound showed a thickened and edematous gall bladder wall with no gallstones or biliary duct dilation (Figure 1). Repeat abdominal and pelvic CT scans showed edematous gall bladder without evidence of stones or bile duct dilation along with splenomegaly.

**Figure 2:**
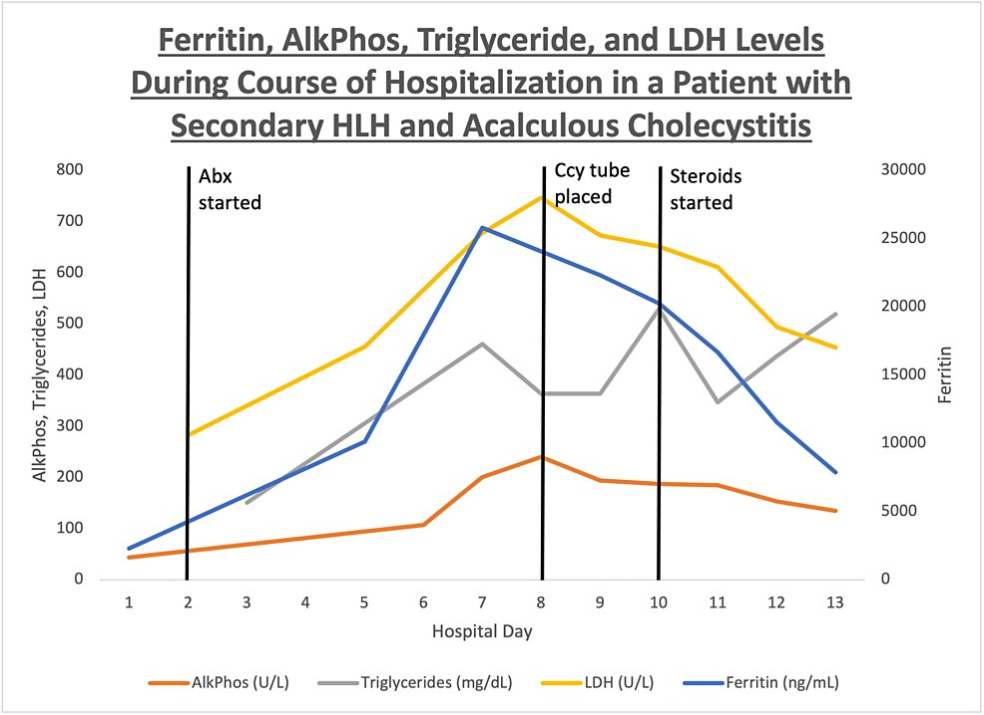
Fever resolved and inflammatory markers were noted to be trending down within 24-48 h of starting steroids. AlkPhos, alkaline phosphatase; LDH, lactate dehydrogenase; Abx, antibiotics; Ccy tube, cholecystostomy tube; HLH, hemophagocytic lymphohistiocytosis

**Figure 3:**
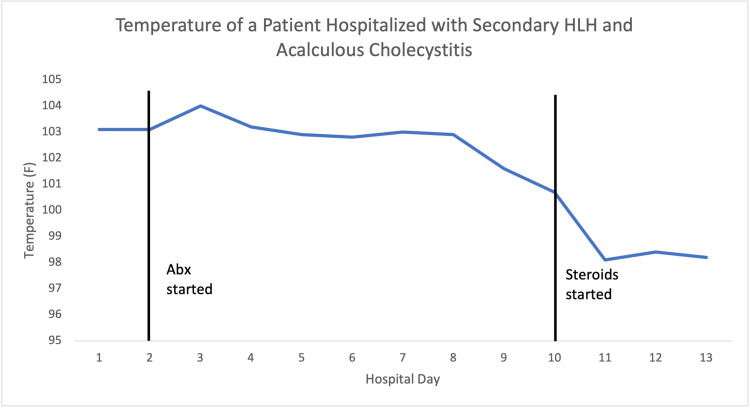
Fever resolved within 24-48 h of starting steroids. HLH, hemophagocyctic lymphohistiocytosis; ABX, antibiotics

Due to continued persistent abdominal pain and fever, a percutaneous cholecystostomy tube was placed with cultures showing no growth. Due to non-responsiveness to antibiotics and persistently elevated inflammatory markers, he was started on dexamethasone (10 mg/m2 daily for two weeks followed by a taper). As shown in Figures 2-3, his fever resolved, and inflammatory markers were noted to be trending down within 24-48 h of starting steroids. His labs on the day of discharge are shown in Table 1. After discharge, he underwent outpatient laparoscopic cholecystectomy, and genetic testing for primary HLH was found to be negative.

## Discussion

Hemophagocytic lymphohistiocytosis is a life-threatening syndrome with a high mortality rate. It is caused by an overactivation and production of proinflammatory cytokines that wreak havoc throughout the body, attacking endothelial tissues and resulting in end-organ destruction [2]. Acquired HLH occurs in autoinflammatory and autoimmune diseases (macrophage activation syndrome) and in patients with iatrogenic immunosuppression or with malignancies, but also in otherwise healthy persons with infections [3]. Our patient was diagnosed with Granulomatosis polyangiitis with renal involvement at age sixteen. He was in remission for the last three years but continued to be on immunosuppressive therapy with azathioprine and prednisone. His history of autoimmune disease and current immunosuppressive therapy increased his risk of developing acquired Hemophagocytic lymphohistiocytosis (HLH). There can be several pathological triggers for it, however, the most common one is an infection, which can be from any bacteria, mycoplasma, fungi, or viruses. They act as continuous, antigens or activators of receptors that further cause uncontrolled activation of cells of the innate immune system. Both sepsis and HLH have several overlapping features [3,4]. It is important for the patient to undergo comprehensive testing of all possible infectious etiologies because if present, treatment of the underlying cause is crucial. Initial infectious workup including several diagnostic tests and imaging studies sent for this patient was negative.

For HLH to be diagnosed five out of eight specified criteria must be met. These include splenomegaly, fever greater than or equal to 38.5OC, cytopenia (with at least two of hemoglobin <9 g/dL, platelets <100,000/microL, or absolute neutrophil count <1,000/microL), low NK cell activity, ferritin >500 ng/mL, hypertriglyceridemia and/or hypofibrinogenemia, elevated soluble IL-2 receptor alpha, or evidence of hemophagocytosis [3]. Our patient met 7/8 criteria including splenomegaly, fever, pancytopenia, low NK cell levels, hyperferritinemia, hypertriglyceridemia, and elevated soluble IL-2 receptor alpha levels. He did not meet the criteria for hemophagocytosis found on either bone marrow, spleen, lymph node, or CSF.

Acute acalculous cholecystitis has been reported in some adult patients with HLH in the past. Excessive immune system activation results in endothelial injury and increased vascular permeability, possibly leading to local microvascular ischemia of the gallbladder wall and bile stasis in AAC [5]. Acute acalculous cholecystitis has been linked with bile stasis, ischemia and reperfusion injuries, total parenteral nutrition, infection, positive-pressure ventilation, and more [6]. Several cases have been reported in the children. Similar pathology including endothelial damage, ischemia, and stasis leading to higher concentrations of bile salts was seen in children presenting with AAC. Most likely, the hypo-perfused state in critically ill children can increase the risk of AAC [7]. However, we were able to review four such cases in adults [5,8,9,10]. Two of them with underlying autoimmune diseases, adult-onset Still’s disease and Kikuchi disease [5, 8]. Both of these patients unlike ours were female and presented with high disease activity and recurrence. This might have been a trigger to cause HLH and acalculous cholecystitis. Our male patient did not show any signs of a flare of granulomatosis polyangiitis with baseline kidney function, no hematuria, no upper or lower respiratory tract symptoms, and a PR3 level of zero. However, similar to our patient, definitive treatment was achieved with both steroids and immunosuppressive medications [5, 8].

In this patient, it is unclear if acute cholecystitis developed first and was a potential trigger for developing HLH, or intense immune activation and cytokine storm of HLH caused gallbladder inflammation. Since he did not respond to antibiotics and the clinical picture continued to worsen, it is suspected that acute acalculous cholecystitis developed due to an intense inflammatory response activated in the body from HLH. Elevation of liver enzymes and hyperbilirubinemia with possible abdominal symptoms should be a sign of gallbladder inflammation in patients who are meeting HLH criteria. If acute cholecystitis could be a potential trigger of HLH, antibiotics should be started and tapered based on cultures. However, early initiation of immunosuppressive therapy under careful observation can reduce mortality and can ultimately control the activated immune/ cytokine pathway and improve gallbladder inflammation [5, 8]. This is also superior to early surgical intervention including drain placement and cholecystectomy, as evident in our reported case and other prior four cases. Therefore, these procedures should not delay the start of immunosuppressive therapy, which can be potentially fatal. 

## Conclusions

Hemophagocytic lymphohistiocytosis can develop at any time during the course of rheumatological disorders, including flare, and remission if the patient is on immunosuppressive therapy and in the presence of concurrent infection. HLH and sepsis have several overlapping features, therefore comprehensive infectious workup is recommended. Abdominal pain with or without elevated liver function tests should raise suspicion of acalculous cholecystitis in patients with HLH. This can cause a further increase in mortality. Starting the steroids as per HLH protocol should be prioritized. This will be lifesaving without the need for any urgent surgical procedures.

## References

[1] Owen CC, Jain R (2005). Acute acalculous cholecystitis. Curr Treat Options Gastroenterol.

[2] Memon F, Ahmed J, Malik F, Ahmad J, Memon DA (2020). Adult-onset primary hemophagocytic lymphohistiocytosis: reporting a rare case with review of literature. Cureus.

[3] Janka GE, Lehmberg K (2013). Hemophagocytic lymphohistiocytosis: pathogenesis and treatment. Hematology Am Soc Hematol Educ Program.

[4] Machowicz R, Janka G, Wiktor-Jedrzejczak W (2017). Similar but not the same: Differential diagnosis of HLH and sepsis. Crit Rev Oncol Hematol.

[5] Otsuka Y, Inoue Y (2016). So-called acute acalculous cholecystitis in macrophage activation syndrome. Intern Med.

[6] Barie PS, Eachempati SR (2003). Acute acalculous cholecystitis. Curr Gastroenterol Rep.

[7] Poddighe D, Tresoldi M, Licari A, Marseglia GL (2015). Acalculous acute cholecystitis in previously healthy children: general overview and analysis of pediatric infectious cases. Int J Hepatol.

[8] Arai Y, Ishikawa Y, Abe K, Kato Y, Abe D, Fujiwara M, Kita Y (2021). A recurrent case of adult-onset Still's disease with concurrent acalculous cholecystitis and macrophage activation syndrome/hemophagocytic lymphohistiocytosis successfully treated with combination immunosuppressive therapy. Intern Med.

[9] Brambilla B, Barbosa AM, Scholze CD (2020). Hemophagocytic lymphohistiocytosis and inflammatory bowel disease: case report and systematic review. Inflamm Intest Dis.

[10] Manasa K, Stosh E, Umair A, Jacqueline F (2017). Secondary hemophagocytic lymphohistiocytosis presenting as acalculous cholecystitis. Am J Gastroenterol.

